# Association Between Asian Flush and Satisfaction of Sleep via Alcohol Consumption Status in a Sample of Japanese Participants

**DOI:** 10.3390/medsci12040062

**Published:** 2024-11-08

**Authors:** Yuji Shimizu, Tomokatsu Yoshida, Keiko Ito, Kumiko Terada, Nagisa Sasaki, Eiko Honda, Kazushi Motomura

**Affiliations:** 1 Epidemiology Section, Division of Public Health, Osaka Institute of Public Health, Osaka 537-0025, Japan; tmyosida@iph.osaka.jp (T.Y.); keitou@iph.osaka.jp (K.I.); terada@iph.osaka.jp (K.T.); sasaki@iph.osaka.jp (N.S.); honda@iph.osaka.jp (E.H.); motomura@iph.osaka.jp (K.M.); 2 Department of General Medicine, Nagasaki University Graduate School of Biomedical Sciences, Nagasaki 852-8501, Japan; 3 Department of Public Health, Osaka University Graduate School of Medicine, Osaka 565-0871, Japan

**Keywords:** alcohol, Asian flush, quality of sleep, sleep

## Abstract

Background: The reddening of the face and neck following alcohol consumption is known as Asian flush. Although genetic factors related to Asian flush have been reported to be inversely associated with duration of sleep, no study has reported an association between Asian flush and sleep satisfaction. Methods: A cross-sectional study of 3823 Japanese people, aged 20 to 64 years was conducted. The target population comprised general public resident monitors of Osaka Prefecture who were registered with an internet research company. Results: A significant inverse association was observed between Asian flush and satisfaction of sleep. The potential confounder-adjusted odds ratio (OR) and 95% confidence interval (CI) of satisfied sleep was 0.81 (0.69–0.96). The alcohol consumption status-specific analysis revealed essentially the same associations between consumers and non-consumers of alcohol. The adjusted ORs (95% CIs) were 0.81 (0.65–0.997) for non-consumers and 0.80 (0.61–1.05) for consumers of alcohol. Conclusion: The genetic characteristics of physical reactions to alcohol exposure may influence sleep quality. One’s alcohol consumption status may not influence the effects of having a lower tolerance to alcohol on sleep quality.

## 1. Introduction

Asian flush is an alcohol–flush reaction, which is a characteristic physical reaction, such as the reddening of the face and neck, following alcohol consumption. Approximately 36% of the East Asian population has characteristics of Asian flush [1].

Genetic factors that influence Asian flush [2] are associated with unusual sleep duration, possibly due to alcohol consumption [3]. Although there are individual differences in the optimal duration of sleep [4], Asian flush may be associated with poor quality of sleep. However, no studies have reported an association between Asian flush and satisfaction of sleep.

A recent study that evaluated trends in sleep problems between 2004 and 2017 revealed that insomnia and poor quality of sleep decreased, while shorter sleep duration and late bedtime increased [5]. In 2004, the Japanese had one of the shortest sleep durations, worldwide [6]. If the genetic characteristics of Japanese people are causing poor quality of sleep, the present trend is critical for Japanese society, particularly for workers.

Furthermore, unlike other nations, people in Japan tend to consume alcohol when they have difficulty sleeping [6]. Therefore, an alcohol consumption status-specific analysis on the association between Asian flush and satisfaction of sleep is necessary to identify the mechanism underlying the association between Asian flush and satisfaction of sleep.

Hence, to evaluate the association between physical reactions to alcohol exposure and the influence of alcohol consumption status on sleep quality, a cross-sectional study of 3823 people who participated in a web-based survey was conducted.

## 2. Materials and Methods

### 2.1. Study Population

A web-based survey was conducted from 27 to 30 November 2023, to identify the proportion of the general public with developmental disorders and/or psychiatric disorders, as several individuals with such disorders may have difficulty participating in social services such as annual health checkups.

The target population comprised individuals aged 20–64 years among general public resident monitors of Osaka Prefecture who were registered with Cross Marketing, Inc. (Tokyo, Japan), an internet research company.

Initially there were 3865 participants (1926 males and 1939 females) aged 20 to 64 years. To avoid the influence of severe sleep disorders and reflect daily life, participants who sleep for more than 18 h per day (n = 10) or less than two hours per day (n = 32) were excluded. Finally, there were 3823 participants (1907 males and 1916 females) with a mean ± standard deviation (sd) of 43.7 ± 12.2 years. The mean age for males was 44.2 ± 12.0 years and the mean age for females was 43.2 ± 12.5 years.

During the survey, an e-mail was sent to all the resident monitors requesting informed consent to participate. This study was approved by the Ethics Committee of the Osaka Institute of Public Health (Project registration code: 2307-01).

### 2.2. Definitions

The participants were asked to select their status from the following options: “satisfied”, “slightly dissatisfied”, “quite dissatisfied”, or “very dissatisfied or I couldn’t sleep at all”. Those who responded with “satisfied” were considered to have satisfactory sleep.

Since self-reported alcohol flushing can serve as an instrumental variable for evaluating alcohol consumption [7], we defined Asian flush using a questionnaire in this study. The participants were classified as experiencing Asian flushing if they responded “yes” to the question “Does a small amount of alcohol intake (about 1 glass of beer) make your face turn red immediately at the age when you first started drinking alcohol?”.

The Kessler psychological distress scale (K6) screens for severe mental illness, defined as a K6 score ≥ 13 [8]. The participants who have never consumed alcohol and the former consumers of alcohol were defined as non-consumers. Walking was defined as spending more than 10 min walking more than three times a week. Exercise was defined as performing strenuous physical activity (e.g., carrying heavy loads, cycling uphill, jogging, or singles tennis) more than once a week. Based on previous studies, including a Japanese study that defined normal sleep duration as 6–8 h [9,10], short sleep was defined as those sleeping for less than 6 hours, and long sleep was defined as those sleeping for more than 8 h.

### 2.3. Statistical Analysis

The characteristics of the study were presented as percentages except for age. Logistic regression models were used to calculate odds ratios (ORs) and 95% confidence intervals (CIs) to determine the association between satisfactory sleep and Asian flush.

Three adjusted models were used to evaluate the association between satisfactory sleep and Asian flush, for overall and sex-specific analyses. Model 1 was adjusted for sex and age. Model 2 was adjusted for alcohol consumption status, mental illness, walking, and exercise, in addition to sex and age. Model 3 was additionally adjusted for short sleep and long sleep, in addition to the factors in Model 2.

For the analyses between satisfactory sleep and Asian flush via alcohol consumption status, three adjusted model were also used. Model 1 was adjusted for sex and age. Model 2 was adjusted for mental disease, walking, and exercise, in addition to sex and age. Model 3 was additionally adjusted for short sleep and long sleep, in addition to the factors in Model 2.

All the statistical analyses were performed using SAS for Windows (version 9.4; SAS Inc., Cary, NC, USA). The statistical significance was set at *p* < 0.05.

## 3. Results

### 3.1. Clinical Characteristics of Study Cohort by Asian Flush Status

The Asian flush-specific clinical characteristics of the study cohort are shown in Table 1. Compared to participants without Asian flush, those with Asian flush were significantly older and had a higher prevalence of having mental illness, having waking habits, and being short sleepers, and a lower prevalence of being current consumers of alcohol, having exercise habits, and being long sleepers.

### 3.2. Association Between Asian Flush and Satisfaction of Sleep

Table 2 shows the association between Asian flush and satisfaction of sleep. For the overall and the male participants, independent of potential confounders, Asian flush was significantly inversely associated with satisfaction of sleep and for females, the association was not statistically significant. The fully adjusted ORs (95% CIs) of satisfaction of sleep for Asian flush were 0.81 (0.69–0.96) for the overall cohort, 0.77 (0.61, 0.98) for males, and 0.84 (0.66, 1.06) for females.

### 3.3. Association Between Asian Flush and Satisfaction of Sleep via Alcohol Consumption Status

Table 3 shows the alcohol consumption status-specific associations between Asian flush and satisfaction of sleep. For non-consumers of alcohol and consumers of alcohol, in Model 1 and Model 2, a significant inverse association between Asian flush and satisfaction of sleep was observed. Among non-consumers of alcohol, even after additional adjustment for short sleep and long sleep, the association remained statistically significant. However, among consumers of alcohol the association was not statistically significant. The fully adjusted ORs (95% CIs) of satisfaction of sleep for Asian flush were 0.81 (0.65, 0.997) for non-consumers of alcohol and 0.80 (0.61, 1.05) for consumers of alcohol.

## 4. Discussion

The major finding of this study is that Asian flush, which is related to low tolerance for alcohol, is significantly inversely associated with satisfaction of sleep and this inverse association was also observed when the analysis was limited to non-consumers of alcohol.

This is the first study to report an inverse association between Asian flush and satisfaction of sleep, even among non-consumers of alcohol.

An inherited deficiency of the enzyme aldehyde dehydrogenase 2 (ALDH2) is the predominant cause of Asian flush [2].

A previous study of 5140 Japanese people reported that the functional ALDH2 variant (rs671) strongly influenced the usual duration of sleep, possibly due to alcohol consumption [3]. That study is partially compatible with the present findings, which show that people with Asian flush have significantly shorter duration of sleep than those without Asian flush. However, in the present study, an inverse association between Asian flush and sleep satisfaction was also observed when the analysis was limited to non-consumers of alcohol.

Therefore, independent of alcohol consumption, ALDH2 variant (rs671)-related factors may influence the association between Asian flush and satisfaction of sleep.

Strong linkage disequilibrium values between rs3782886 (breast cancer suppressor BRCA1-related associated protein [BRAP]) and rs671 (ALDH2) have been reported [11]. Almost all the participants with a genetic variant of ALDH2 had genetic characteristics of the BRAP variant [12].

In atherosclerotic lesions, BRAP is expressed in macrophages and smooth muscle cells [13]. BRAP activates an inflammatory cascade by activating [14] nuclear factor-κB (NF-κB) [15] and the BRAP-related inflammatory response is independent of alcohol consumption [12,16].

Since sleep enhances immune defenses, and afferent signals from immune cells promote sleep [17], chronic inflammation may increase the requirement for sleep, which may result in dissatisfied sleep, even with a normal duration of sleep. However, in the present study, among non-consumers of alcohol, the prevalence of short sleepers among those with Asian flush (27.3%) was significantly higher than those without Asian flush (22.3%, *p* = 0.008). Furthermore, the prevalence of long sleepers among non-consumers of alcohol with Asian flush (4.1%) was significantly lower than those without Asian flush (7.0%, *p* = 0.004). Therefore, the physical characteristics of Asian flush may shorten duration of sleep. However, even after additional adjustment for short sleep and long sleep, a significant inverse association between Asian flush and satisfaction of sleep was observed.

Therefore, the physical characteristics of easy-to-activate inflammation may disturb not only the duration of sleep but also the quality of sleep. People with sleep problems are at greater risk of developing immune and chronic inflammatory diseases [18]. Thus, variants of BRAP may underlie the association between Asian flush and satisfaction of sleep.

This study had several novel clinical implications. First, a questionnaire that is easy to introduce in daily clinical practice and annual health check-ups could act to identify risk factors for sleep deprivation. Second, even among non-consumers of alcohol, Asian flush was revealed as a risk for sleep disturbance, not only regarding duration of sleep but also satisfaction of sleep.

There are limitations to this study. First, since the data were obtained from a web-based questionnaire, selection bias could not be eliminated. Second, the activation of an inflammatory pathway related to NF-κB might be underlying the results; however, there are no data related to NF-κB. To clarify the mechanism underlying the results, determining the NF-κB status is required. Finally, the alcohol consumption status-specific analysis did not reach statistical significance. However, essentially the same associations were observed for those who have never consumed alcohol, former consumers of alcohol, and current consumers of alcohol.

## 5. Conclusion

A significant inverse association was observed between Asian flush and satisfaction of sleep. The genetic characteristics of physical reactions to exposure to alcohol may influence sleep quality. Alcohol consumption status may not influence the effect of lower tolerance to alcohol on quality of sleep.

## Figures and Tables

**Table 1:** Characteristics of study cohort by Asian flush status.

	Asian Flush		*p*
	(No)	(Yes)	
Participants, n	2431	1392	
Males, %	50.6	48.6	0.217
Age, years	42.9 ± 12.2	45.1 ± 12.2	<0.001
Current consumer of alcohol, %	51.5	32.7	<0.001
Former consumer of alcohol, %	7.1	7.3	0.808
Mental illness, %	6.3	17.8	<0.001
Exercise, %	39.0	26.3	<0.001
Walking, %	44.7	51.1	<0.001
Short sleep, %	21.8	26.9	<0.001
Long sleep, %	5.7	3.6	0.004

Ages are expressed as mean ± standard deviation.

**Table 2:** Association between satisfactory sleep and Asian flush.

	Asian Flush		*p*	*p* (Goodness of Fit Test)
	(No)	(Yes)		
Overall				
Participants, n	2431	1392		
Satisfactory sleep, n (%)	631 (26.0)	296 (21.3)		
Model 1	Reference	0.78 (0.67, 0.91)	0.002	0.731
Model 2	Reference	0.77 (0.65, 0.90)	0.002	0.935
Model 3	Reference	0.81 (0.69, 0.96)	0.012	0.412
Males				
Participants, n	1231	676		
Satisfactory sleep, n (%)	326 (26.5)	138 (20.4)		
Model 1	Reference	0.72 (0.58, 0.91)	0.005	0.959
Model 2	Reference	0.72 (0.57, 0.91)	0.006	0.836
Model 3	Reference	0.77 (0.61, 0.98)	0.030	0.968
Females				
Participants, n	1200	716		
Satisfactory sleep, n (%)	305 (25.4)	158 (22.1)		
Model 1	Reference	0.84 (0.67, 1.04)	0.114	0.781
Model 2	Reference	0.82 (0.65, 1.02)	0.080	0.593
Model 3	Reference	0.84 (0.66, 1.06)	0.143	0.992

Model 1: adjusted only for sex and age. Model 2: in addition to sex and age, adjusted for alcohol consumption status, mental illness, walking, and exercise. Model 3: in addition to factors in Model 2, adjusted for short sleep and long sleep.

**Table 3:** Association between satisfactory sleep and Asian flush by alcohol consumption status.

	Asian Flush		*p*	*p* (Goodness of Fit Test)
	(No)	(Yes)		
Non-consumer of alcohol				
Participants, n	1179	937		
Satisfactory sleep, n (%)	314 (26.6)	204 (21.8)		
Model 1	Reference	0.77 (0.63, 0.95)	0.014	0.416
Model 2	Reference	0.77 (0.62, 0.95)	0.013	0.943
Model 3	Reference	0.81 (0.65, 0.997)	0.047	0.995
Consumer of alcohol				
Participants, n	1252	455		
Satisfactory sleep, n (%)	317 (25.3)	92 (20.2)		
Model 1	Reference	0.75 (0.58, 0.97)	0.029	0.190
Model 2	Reference	0.76 (0.58, 0.99)	0.040	0.782
Model 3	Reference	0.80 (0.61, 1.05)	0.108	0.523

Model 1: adjusted only for sex and age. Model 2: in addition to sex and age, adjusted for mental illness, walking, and exercise. Model 3: in addition to factors in Model 2, adjusted for short sleep and long sleep.

## Data Availability

We cannot publicly provide individual data due to the participants’ privacy, according to ethical guidelines in Japan. Additionally, the informed consent that was obtained did not include a provision for publicly sharing data. Qualifying researchers may apply to access a minimal dataset by contacting Dr Yuji Shimizu, Principal Investigator, Epidemiology Section, Division of Public Health, Osaka Institute of Public Health, Osaka, Japan at simizu@iph.osaka.jp, or, by contacting the office of data management at epiana@iph.osaka.jp. Information for where to find data requests is also available at https://www.iph.osaka.jp/s016/index.html (accessed on 3 October 2024).
